# Tilt training as a treatment for reflex syncope: a multimodal approach!?

**DOI:** 10.3389/fnins.2024.1473687

**Published:** 2024-11-29

**Authors:** Miek Hornikx, Peter Haemers, Linda Stans, Tomas Robyns, Christophe Garweg, Joris Ector, Bert Vandenberk, Rik Willems

**Affiliations:** ^1^Department of Physical Medicine and Rehabilitation, Katholieke Universiteit Leuven, Leuven, Belgium; ^2^Department of Cardiovascular Sciences, Katholieke Universiteit Leuven, Leuven, Belgium; ^3^Department of Cardiology, UZLeuven, Leuven, Belgium; ^4^Department of Pneumology, UZLeuven, Leuven, Belgium

**Keywords:** tilt training, tilt test, reflex syncope, presyncope, hyperventilation

## Abstract

**Purpose:**

Reflex syncope is a burdensome disease with considerable repercussions on the quality of life. Tilt training is a therapeutic option, but evidence on this topic is scarce and outdated. Hyperventilation is oftentimes associated with reflex syncope. This study aimed to evaluate the effectiveness of tilt training in patients with reflex syncope and study the association between reflex syncope and hyperventilation.

**Methods:**

Patients referred for tilt training after a positive tilt test from July 2014 to March 2021 were included in a single-center, retrospective registry. Demographic characteristics and outcomes of the program were collected. The response of tilt training on (pre)syncope recurrence and the association with hyperventilation were studied.

**Results:**

A total of 173 patients were included. The median age was 27 [17–48] years. Patients needed 2 [1–3] sessions to reach the first negative tilt training. The tilt training program was successfully completed by 65% of patients. An additional 3% reported no complaints in daily life, despite remaining symptomatic during tilt training. Another therapy was initiated in 10% of patients, while 21% dropped out of the tilt training. Presyncope recurred in 21% of patients during a follow-up period of 21 months [16–23]. Concomitant hyperventilation was suspected in 24%. Among these patients, 74% were referred for a hyperventilation provocation test, which confirmed the diagnosis in 82%.

**Conclusion:**

We report a reasonable success of tilt training in a contemporary cohort of patients. In patients completing the tilt training program, presyncope, and syncope recurrence was low. Concomitant hyperventilation seems prevalent in patients with reflex syncope and warrants specific attention and treatment.

## 1 Introduction

Reflex syncope (RS) is the most common, non-traumatic cause of transient loss of consciousness. RS is caused by cerebral hypoperfusion, due to a sudden temporary failure of the autonomic nervous system to maintain adequate blood pressure and heart rate. Despite RS being a benign condition, the impact on the quality of life of patients is significant (Ng et al., 2019). Furthermore, clinical expertise has led to the realization that reflex syncope is a multimodal condition, involving fear of syncope associated with hyperventilation (HV). HV is considered dysfunctional breathing leading to hypocapnia, typically resulting in a decrease in cerebral blood flow and possibly (pre)syncope (Vidotto et al., 2019). Among HV, two types can be differentiated (Pfortmueller et al., 2015). In primary HV, HV is considered the cause of syncope, and the hemodynamic characteristics during tilt training remain stable. In secondary HV, a vicious circle is initiated, consisting of presyncopal symptoms, aggravation of symptoms due to fear of syncope, and eventually syncope.

European guidelines recommend education (class I), lifestyle modification (class I), and physical counter-pressure maneuvers (PCMs) (class IIa) as the most important treatment components in patients with RS. Tilt training may be considered (class IIb) (Brignole et al., 2018). This class IIb recommendation is based on older evidence (Tan et al., 2010; Duygu et al., 2008; Foglia-Manzillo et al., 2004; Reybrouck et al., 2002). Tilt training may appear unattractive to patients as it is time-consuming, requires a one-on-one approach, and may provoke symptoms associated with presyncope (Foglia-Manzillo et al., 2004). On the other hand, it is non-invasive, free of complications, and backed by evidence demonstrating its usefulness in older patient series. Therefore, the correct referral for this treatment is essential to maximize patient benefit. Treatment of HV typically consists of breathing exercises or cognitive behavioral therapy (Folgering, 1999). As such, patients with RS and HV may require a different approach, for example, a combination of tilt training and breathing exercises. Therefore, it is important to have more accurate knowledge of the prevalence of HV in patients with RS and to investigate the characteristics of these patients.

We aimed to investigate the current effectiveness of tilt training in patients with RS, including the number of sessions until the first negative tilt training and the overall success of the tilt training program. Furthermore, we investigated the suspicion of concomitant HV during RS and described the characteristics of patients with (suspected) HV and the effects on the success of tilt training.

## 2 Materials and methods

### 2.1 Study design and population

All patients with clinical suspicion of RS who had a positive diagnostic tilt test and who were referred for tilt training between July 2014 and March 2021 were included in a single-center, retrospective registry. Patients without a positive diagnostic tilt test or patients referred for tilt training without the availability of the detailed results of the tilt test were excluded from the study. The study was approved by the local Ethics committee (S65888). Given the retrospective design, the need for informed consent was waived.

Patient demographics, clinical characteristics, and medication profiles were collected by reviewing the electronic medical records. When describing the medication, etilefrine hydrochloride and fludrocortison were classified as medication for chronic hypotension. Anti-depressants were subdivided into selective reuptake inhibitors, non-selective reuptake inhibitors, and anti-depressants directly working on a neuroreceptor. Benzodiazepines were categorized as hypnotics, sedatives, and anxiolytics. Benzamides and atypical anti-psychotics were considered anti-psychotics.

### 2.2 Tilt test

For the diagnostic tilt test, patients were in a fasted state and a venous cannulation was placed before starting the test. Straps were placed around the knees and the chest of patients. After baseline measurements, patients were positioned against a table at 60° according to the Westminster protocol (Reybrouck et al., 2002). Heart rate and heart rhythm were registered continuously. Blood pressure was measured every 2.5 min. The test was interrupted when a severe symptomatic presyncope or a syncope occurred. Otherwise, the test was continued for 45 min. We used the VASIS classification to subdivide the presenting syncope at diagnostic tilt testing into type I (mixed type), type II (cardioinhibitory type (IIa: without asystole and IIb: with asystole), and type III (vasodepressor type) (Brignole et al., 2000). The tilt test was performed without the interference of drugs.

### 2.3 Tilt training

During tilt training, patients were positioned in the same way as during the tilt test without the placement of an intravenous line. Heart rate and heart rhythm were registered continuously. Blood pressure was measured every 5 min. The tilt training was continued until 45 min was reached or until symptoms of (severe) presyncope appeared, and hemodynamic changes could be noticed. During tilt training sessions, full syncope occurrence was avoided as much as possible. Supervised tilt training was provided once a week, and patients were instructed to perform two home sessions of 30 min on a weekly basis. After successful training, patients were followed after 6 weeks, 3 months, 6 months, and 1 year. If, during one of those visits, patients were unable to persevere 45 min, they were advised to restart the full training schedule.

Patients were considered to have completed the tilt training program successfully when (1) patients with a first positive tilt training were able to persevere 45 min in tilted position during three consecutive tests with a weekly test frequency; (2) patients with a first negative tilt training and a second positive tilt training at 6-week follow-up were able to persevere 45 min in a tilted position during 3 consecutive weeks; and (3) patients with an initial negative tilt training achieved a second negative training at 6-week follow-up. If patients remained symptomatic during tilt training but were no longer symptomatic in daily life, the tilt training was considered to have been clinically successful. The tilt training was considered not to have been successful if the patients were referred for another treatment, such as relaxation, breathing exercises, psychological consultation, and treatment for Huntington's disease. Drop-outs were defined as patients not showing up for tilt training or not planning follow-up appointments.

### 2.4 Hyperventilation

If the technician performing the tilt training suspected hyperventilation based on respiratory patterns observed during the training or the patient's history of daily life episodes of presyncope, the patient was referred for a hyperventilation provocation test (HVPT) to diagnose hyperventilation (HV). This test was performed in combination with a hyperventilation symptom-specific questionnaire (Nijmegen questionnaire) and a psychological consultation in the pneumology department. The diagnosis of hyperventilation was considered likely when three out of four criteria were met:

- a score of ≥18 on the Nijmegen questionnaire.
- reproducibility of prodromal symptoms during HVPT.
- resting end-tidal carbon dioxide tension (PETCO_2_) < 32 mmHg or fluctuation in resting PETCO_2_ > 2 mmHg during the adaptation phase.
- (resting PETCO_2_/PETCO_2_ value after 3 min recovery) ≥1.5 during the recuperation phase (Hardonk, 1979).

#### 2.4.1 Hyperventilation provocation test

During HVPT, end-tidal CO_2_ levels were measured using a capnometer (Capnostream 20 – Oridion, Medtronic, Belgium). The HVPT itself consisted of three phases: the adaptation phase, voluntary hyperventilation, and recuperation phase. The adaptation phase consisted of 5 min of normal quiet breathing, followed by the voluntary hyperventilation phase for 3 min. A fast (60 breaths per minute) and deep breathing pattern on the rhythm of a metronome was stimulated. During the last phase, the recuperation phase, the patient was asked to resume his normal breathing for 5 min (Meuret et al., 2005).

#### 2.4.2 Nijmegen questionnaire

The Nijmegen questionnaire (NQ) consists of 16 possible symptoms or complaints, scored on a 4-point Likert scale (1 = seldom, 4 = very often), that the patient could have experienced while suffering from HV. The complaints take into account all the different systems (respiratory, cardiovascular, neurological, gastrointestinal, and psyche). A summation was made of all scores. A score of 18 or higher indicated that HV was possible. The higher the score, the higher the likelihood. A score of 23 or higher was very likely (80%) to be positive for HV (Van Dixhoorn and Duivenvoorden, 1985).

#### 2.4.3 Psychological consult

During a consult with a psychologist, the results of the NQ and the HVPT were discussed. The psychologist screened the patient's psychological-, social- and physical wellbeing, significant life events, and internal (personality characteristics) or external (professional/relational…) stressors. With this screening, the psychologist tried to discover underlying problems and mechanisms that could trigger or maintain the HV response. Thereafter, the psychologist reported on the diagnosis of HV, provided psycho-education, and proposed therapy tailored to the patient's profile. Therapy advice consisted mainly of breathing exercises, relaxation therapy, and/or cognitive behavior therapy (CBT). If patients were diagnosed with HV and received a recommendation for treatment, we recommended continuation of the tilt training program in combination with HV treatment.

### 2.5 Statistical analyses

SAS Enterprise Guide 8.2 was used to perform the statistical analyses. A Shapiro–Wilk test was performed on continuous variables to check the normality of data. If a normal distribution was present, the data were presented as mean ± standard deviation. Otherwise, the data were presented using medians and quartiles. A Kruskal–Wallis test was performed to compare the groups based on the type of RS and hyperventilation. Categorical variables were presented as numbers and corresponding percentages. A comparison of categorical variables was performed using Fisher's exact test. A *p*-value of < 0.05 was considered statistically significant.

## 3 Results

### 3.1 Demographic characteristics

**Figure 1:**
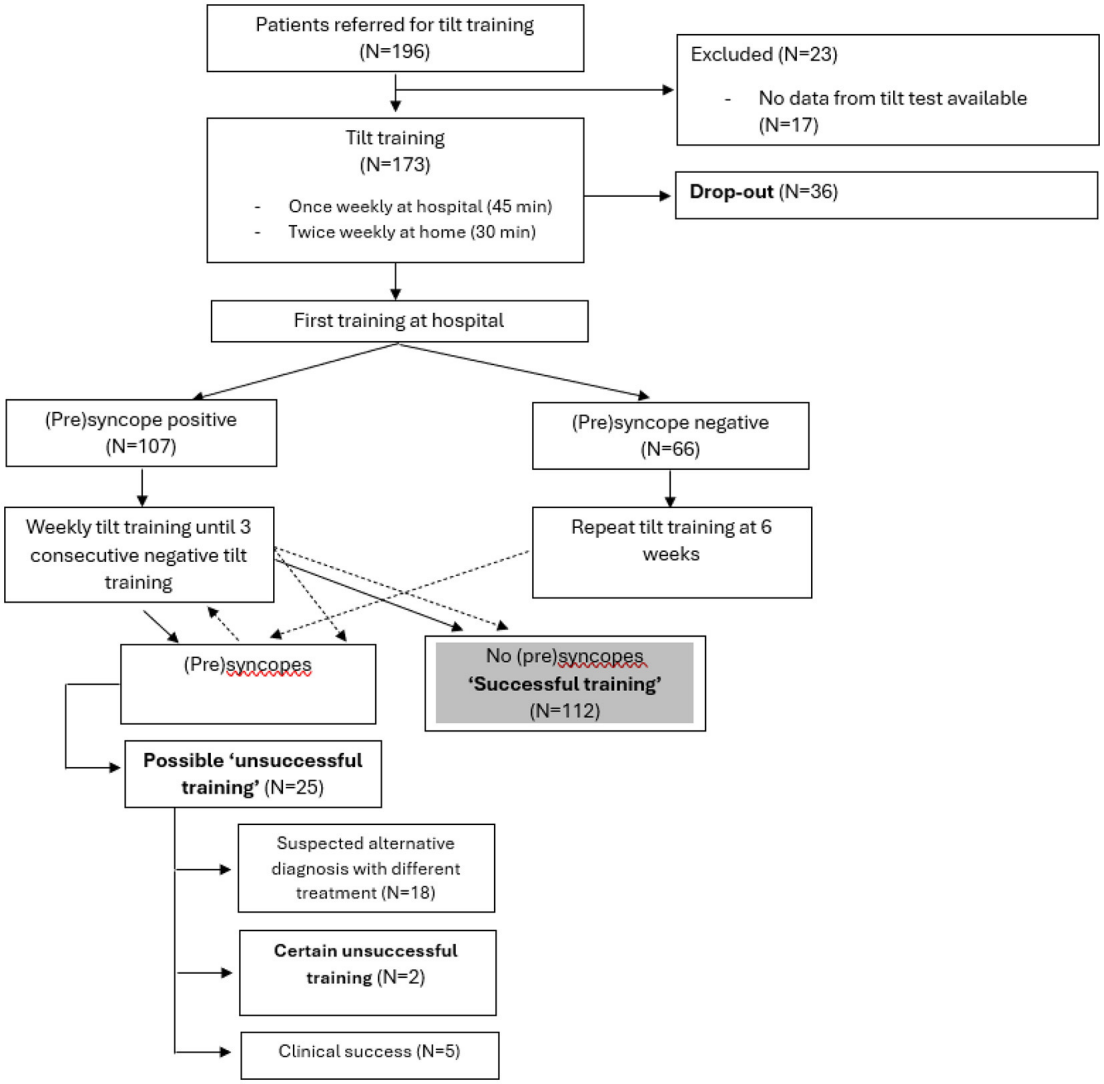
Flowchart of the study.

**Figure 2:**
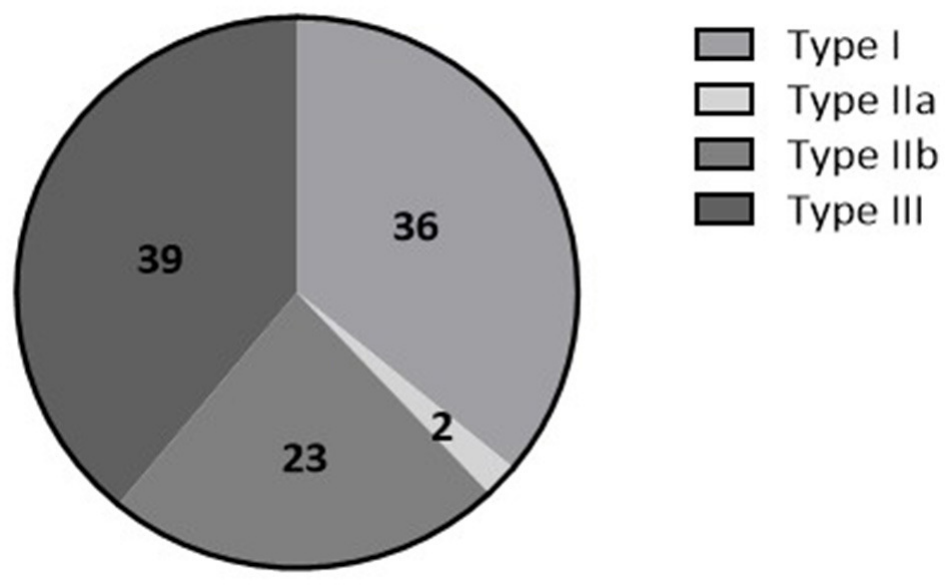
Percentage of patients having Type I (mixed (pre)syncope), Type IIa (cardioinhibitory type without asystole), Type IIb (cardioinhibitory type with asystole), or type III (vasodepressor type) there is stated of RS based on the VASIS classification (Brignole et al., 2000).

Between July 2014 and March 2021, 196 patients were included in the tilt training program. Among these, 173 patients (27 [17–48] years, 73% female) performed an initial positive diagnostic tilt test and were thereafter referred for tilt training. In the remaining 23 patients, either the diagnostic test was negative (*n* = 6) or the detailed tilt test results were not available for analysis (*n* = 17) (Figure 1). The majority of patients were classified within VASIS type I or type III RS (Figure 2). The median asystole duration in type IIb RS was 12 s [7–24]. The rhythm during asystole was most often sinus pause (97%) (Table 1). Other demographic characteristics were not different among the types of RS.

**Table 1:** Demographic characteristics.

	**N = 173**	**Possibly unsuccessful (N = 25)**	**Drop-out (N = 36)**	**Successful (N = 112)**	P-value^*^
Age (years)	27 [17–48]	18 [14–33]	25 [16–42]	31 [18–54]	0.03
Age < 18 years	47 (27)	12 (48)	11 (31)	24 (21)	0.02
Female	126 (73)	20 (80)	30 (83)	76 (68)	0.16
Type of RS					0.36
- Type I	63 (36)	8 (32)	16 (44)	39 (35)	
- Type IIa	3 (2)	0 (0)	2 (6)	1 (1)	
- Type IIb	40 (23)	6 (24)	5 (14)	29 (26)	
- Type III	67 (39)	12 (48)	13 (36)	42 (38)	
Type IIb: duration asystole (seconds)	12 [7–24]	9 [8–9]	26 [25–29]	12 [7–21]	0.33
Duration of diagnostic tilt test (minutes)	22 [13–35]	22 [10–34]	28 [13–37]	21 [12–34]	0.2
Duration of first tilt training (minutes)	35 [16–45]	20 [13–30]	36 [21–45]	37 [17–45]	0.01
Sessions until first negative tilt training (n)	2 [1–3]	2 [1–4]	1 [1–2]	2 [1–4]	0.07
Sessions until 3 × 45 min (n)	7 [5–10]	6 [4–8]	5 [3–9]	7 [5–10]	0.1
**Comorbidities**					
**Cardiac**					
- Heart failure	3 (2)	1 (4)	1 (3)	1 (1)	0.28
- Ischemic heart disease	6 (3)	0 (0)	2 (6)	4 (4)	0.69
Diabetes	4 (2)	0 (0)	1 (3)	3 (3)	1
Depression	19 (11)	3 (12)	4 (11)	12 (11)	0.75
Epilepsy	7 (4)	2 (8)	0 (0)	5 (4)	0.21
**Medication**					
Betablockers	21 (12)	3 (12)	6 (17)	12 (11)	0.54
**Medication chronic hypotension**					
- Etilefrine hydrochloride	11 (6)	2 (8)	2 (6)	7 (6)	0.9
- Fludrocortison	5 (3)	1 (4)	0 (0)	4 (4)	0.65
**Anti-depressants**					
- SSRI	9 (5)	0 (0)	2 (6)	7 (6)	0.53
- NSRI	1 (1)	0 (0)	0 (0)	1 (1)	1
- Other	9 (5)	2 (8)	1 (3)	6 (5)	0.82
Anti-psychotics	5 (3)	1 (4)	2 (6)	2 (2)	0.75
Hypnotics, sedatives, anxiolytics	7 (4)	0 (0)	2 (6)	5 (4)	0.63
Anti-epileptics	7 (4)	2 (8)	0 (0)	5 (4)	0.21
Drop-out	36 (21)				

RS, Reflex syncope; Type I, mixed (pre)syncope; Type IIa, cardioinhibitory syncope without asystole; Type IIb, cardioinhibitory type with asystole; Type III, Vvasodepressor (pre)syncope (Brignole et al., 2000). SSRI, selective serotonin reuptake inhibitor; NSRI, non-selective serotonin reuptake inhibitor; data are expressed as median [Q1–Q3] or as *n* (%).

### 3.2 Succes of tilt training

After a median of 2 [1–3] sessions, patients had their first negative tilt training. A median of 7 [5–10] sessions were needed to be able to stand 3 × 45 min without presyncope (Table 1). The tilt training program was completed successfully in 65% of patients, and an additional 3% had clinical success in daily life but remained symptomatic during tilt training sessions, amounting to a total clinical success of 68%. The training program was not successful in 10%, and those were referred for another treatment. The drop-out rate was 21%, and this occurred after a median of three [1–5] sessions (Figure 1; Table 1).

Regarding the VASIS subtypes, the duration of the diagnostic tilt test was significantly longer in syncope type III compared to types I and II. Furthermore, patients in type III needed to restart tilt training less often compared to the other two VASIS types (Table 2).

**Table 2:** Demographic characteristics based on the VASIS classification (Brignole et al., 2000).

	**Type I (*n =* 63)**	**Type II (*n =* 43)**	**Type III (*n =* 67)**	***p*-value**
Age (years)	25 [17–48]	24 [16–46]	30 [17–56]	0.36
Female	40 (63)	32 (74)	54 (81)	0.09
Duration of diagnostic tilt test (minutes)	21 [12–33]^*^	14 [7–30]^*^	30 [19–40]	**< 0.01**
Duration of first tilt training (minutes)	31 [15–45]	26 [13–45]	37 [21–45]	0.28
Sessions until first negative tilt training (n)	2 [1–4]	2 [1–4]	1 [1–3]	0.22
Sessions until 3 × 45 min (n)	6 [4–12]	7 [5–10]	7 [5–10]	0.84
Objective hyperventilation	12 (19)	10 (23)	13 (19)	0.86
Anti-depressants, anti-psychotics, hypnotics, sedatives, and anxiolytics	7 (11)	3 (7)	10 (15)	0.47
Drop-out [*n* (%)]	16 (24)	7 (17)	13 (20)	0.12
Restart tilt training [*n* (%)]	16 (44)^*^	13 (36)^*^	7 (19)	**0.02**

Type I, Mixed (pre)syncope; Type II, cardioinhibitory syncope; Type III, vasodepressor (pre)syncope. Data are expressed as median [Q1-Q3] or as *n* (%). *p* < 0.05. ^*^Significant difference with type III. Values in bold are significant values.

### 3.3 Change in type of RS

**Figure 3:**
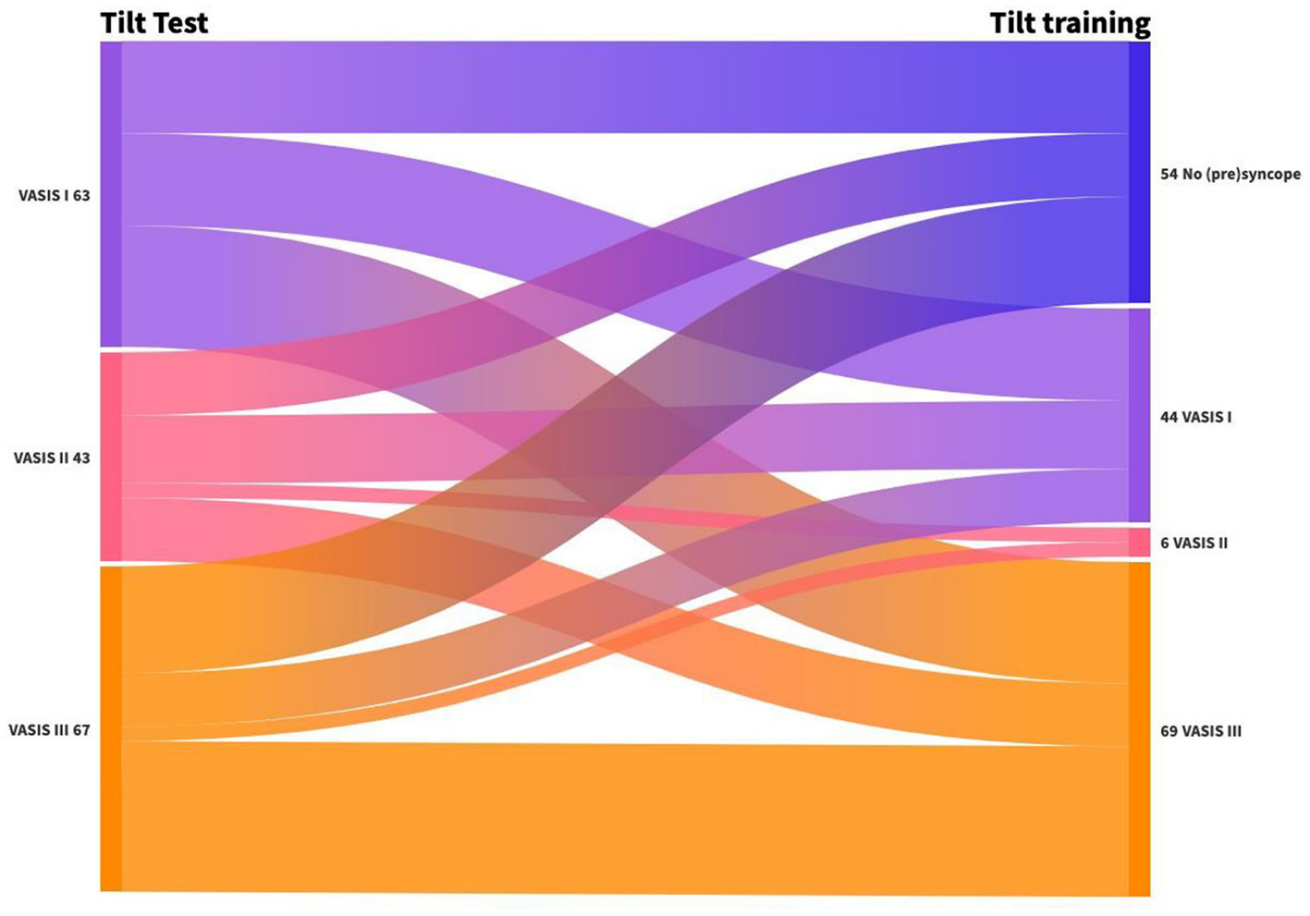
Change in the type of RS from tilt test to first positive tilt training. VASIS I: Mixed (pre)syncope; VASIS II: cardioinhibitory (pre)syncope; and VASIS III: vasodepressor (pre)syncope (Brignole et al., 2000).

Figure 3 depicts the change in the type of RS from the tilt test to the first positive tilt training. We tried to avoid full syncope during training; therefore, severely symptomatic presyncope was considered an endpoint during tilt training. The hemodynamic nature of RS changed frequently between the diagnostic test and the first tilt training: 30% had no syncope, 31% changed in hemodynamic mechanism of (pre)syncope, and 39% demonstrated an identical pathophysiology on the first tilt training. A shift to cardioinhibitory presyncope (VASIS II) was infrequent (3%), while a change to vasodepressor presyncope (VASIS III) was observed in 40%.

### 3.4 (Pre)syncope recurrence

None of the patients needed to restart tilt training because of syncope. Presyncope, however, recurred in 21% of patients. The majority of these patients (94%) had their first positive tilt training (*p* < 0.01). In 97% of patients, tilt training was successful (*p* < 0.01).

### 3.5 Hyperventilation

In 42 patients (24%), hyperventilation was suspected. Among these patients, 31 (74%) performed a HVPT. In 27 (82%) patients, this test confirmed the HV diagnosis. In an additional four (3%) patients, without a suspicion of hyperventilation during tilt training, an HVPT was prescribed by another specialist and was positive. Eight patients (31%) were referred for psychomotor therapy, and fifteen (58%) were encouraged to follow cognitive behavioral therapy. Three patients (12%) were candidates to follow both therapies. The psychological report was not available for one patient. Characteristics of patients with or without hyperventilation are depicted in Table 3. When comparing patients with a suspicion of hyperventilation with patients without hyperventilation, we found a trend toward younger age in the group of patients with a suspicion of hyperventilation. Patients in this group were predominantly female. Patients with a suspicion of hyperventilation had a significantly shorter duration on the first tilt training, needed more sessions to reach a first negative tilt training, and restarted tilt training more often compared to patients without hyperventilation.

**Table 3:** Tilt training outcomes in three groups based on result HVPT.

	**HVobj°(*n =* 35)**	**HVsubj°(*n =* 11)**	**HVsusp^$^ (*n =* 46)**	**No-HV^°*$*^ (*n =* 127)**	***p*-value 3 groups°**	***p*-value 2 groups^$^**
Age (years)	27 [17–36]	19 [14–28]	23 [17–35]	29 [17–56]	0.09	0.05
Female	30 (86)^*^	11 (100)^*^	41 (89)	85 (67)	**< 0.01**	**< 0.01**
Anti-depressants, anti-psychotics, hypnotics, sedatives, and anxiolytics	4 (11)	1 (9)	5 (11)	15 (12)	1.00	1.00
Duration tilt test (minutes)	27 [12–34]	22 [10–38]	25 [12–35]	21 [13–35]	0.86	0.58
Duration of first tilt training (minutes)	21 [13–41]	35 [10–43]	23 [13–41]	36 [17–45]	0.06	**0.02**
Sessions until first negative tilt training (n)	2 [1–4]	3 [1–5]	2 [1–4]	2 [1–3]	0.17	0.06
Sessions until 3 × 45 min (n)	7 [5–9]	11 [9–13]	7 [5–11]	6 [4–10]	0.20	0.30
Drop-out (n (%)	4 (11)	3 (27)	7 (15)	29 (23)	0.29	0.40
Types RS during diagnostic tilt test^#^					0.89	0.83
Type I	12 (34)	3 (27)	17 (37)	48 (38)		
Type IIa	0 (0)	0 (0)	0 (0)	3 (2)		
Type IIb	10 (29)	2 (18)	12 (26)	28 (22)		
Type III	13 (37)	6 (55)	17 (37)	48 (38)		
Restart tilt training [*n* (%)]	9 (26)	4 (36)	13 (28)	23 (18)	0.25	0.06
Successful tilt training [*n* (%)]	21 (60)	4 (36)	25 (54)	87 (69)	0.08	0.11

Data are expressed as median [Q1–Q3] or as *n* (%). HVobj, Patients with suspicion of hyperventilation and a positive hyperventilation provocation test; HVsubj, patients with suspicion of hyperventilation without a hyperventilation provocation test; HVsusp, patients with a suspicion of hyperventilation; No-HV, patients without suspicion of hyperventilation; RS, reflex syncope; Type I, mixed (pre)syncope; Type IIa, cardioinhibitory syncope without asystole; Type IIb, cardioinhibitory syncope with asystole; Type III, vasodepressor (pre)syncope. *p* < 0.05; ^*^significant difference with No-HV group. ^#^Type of RS based on the VASIS classification (Brignole et al., 2000). °Comparison between HVobj, HVsubj, and no-HV. ^$^Comparison between HVsusp en no-HV. Values in bold are significant values.

## 4 Discussion

The results of our study show that 65% of patients completed the tilt training program successfully. Two sessions [1–3] were needed to reach the first negative tilt training. Although none of the patients needed to restart due to syncope, presyncope recurred in 21% of patients. Restarting training due to presyncope was less prevalent in patients with type III RS. Concomitant hyperventilation (HV) with RS was prevalent, and these patients needed more sessions to reach a first negative tilt training and restarted tilt training more often compared to patients without hyperventilation.

### 4.1 Tilt training

The median age of patients was 27 years [17–48]. This is comparable to the reported median age in the former tilt training studies by Reybrouck et al. (2000) and Ector et al. (2005), which showed a median age of 25–27 years and was in line with the fact that RS typically occurs in young patients, particularly before the age of 40 years (Brignole et al., 2018; Goldschlager et al., 2003). Patients in our study were predominantly female (73%), similar to the study of Kenny and McNicholas (2016) and Di Girolamo et al. (1999), who found that the incidence of syncope was higher in women than in men. Most of the patients in our study were classified within the vasodepressor (39%) and mixed type (36%) of RS. The same prevalence of RS was found in the older studies of Reybrouck et al. (2002), Reybrouck et al. (2000), and Ector et al. (2005), in which the majority of patients were classified within the vasodepressor type. In the study of Kinay et al. (2004), 63% of patients with RS were categorized within the mixed type. In terms of the duration of tilt training, two sessions [1–3] were needed to reach the first negative tilt training. This is lower than the mean of three sessions in the study of Reybrouck et al. (2002) A possible explanation might be that patients were older (35 years) in this study, making it more difficult for the reflexes to be trained and to become effective. In the same study, the duration of the tilt test was reported, which was similar in the three types of RS, while we found a significantly longer duration in patients with type III RS. Restart due to presyncope was lower in the latter group. In our experience, patients with type III RS typically get symptoms after approximately 20–30 min of tilt training. These patients benefit the most from education and tilt training.

Reybrouck et al. (2002) assessed the long-term effect of tilt training. They concluded that 82% of patients were syncope-free almost 4 years after following tilt training. We found that 79% of patients were syncope-free after 21 months [16–23] of follow-up. Different tilt training schedules exist, from in-hospital training sessions (Ector et al., 1998) to daily home-based training sessions (Tan et al., 2010; Podd et al., 2015; On et al., 2007). Evidence about home-based training sessions is contradictory. Podd et al. (2015) and On et al. (2007) show that home-based training sessions are ineffective in response to the tilt test or in improving autonomic measures, while Tan et al. (2010) found that 50% were syncope-free at 6 months when doing home-based tilt training.

The mechanism of RS is variable from the tilt test to the first positive tilt training in the same patient. A minority of patients changed to cardioinhibitory presyncope, while 40% changed to vasodepressor presyncope. In the study by Ruiz et al. (1996), 44% of patients exhibited a change in the underlying mechanism of RS between the two tilt tests. In our study, positive results were reproduced in 55% of cases. This result is similar to the study by Aerts et al. (2005), in which the reproducibility of an initial positive diagnostic tilt test was 60%. The reproducibility of an asystolic diagnostic tilt test was found to be poor (Foglia-Manzillo et al., 2002; Omar et al., 2004). Based on these data, we can conclude that a diagnostic tilt test is not ideal to guide therapy and that repeated tilt training might be necessary.

### 4.2 Hyperventilation

Evidence on the prevalence of hyperventilation in patients with RS is scarce. In our study, hyperventilation (HV) was suspected in 24% of patients and conformed in the large majority of these patients, since within this group, 74% performed a hyperventilation provocation test, and 82% of these tested positive. Patients with a suspicion of HV and RS were younger and predominantly female. They had a shorter duration on the first tilt training, needed more sessions to reach a first negative tilt training, and restarted tilt training more often because of presyncope.

We hypothesize that hyperventilation combined with RS requires a multimodal treatment approach, including a more thorough evaluation of HV. This can be performed by monitoring the respiration rate during tilt testing and tilt training and applying the Nijmegen questionnaire, the most widely used instrument to measure the prevalence of hyperventilation (Vidotto et al., 2019; Van Dixhoorn and Duivenvoorden, 1985; Vansteenkiste et al., 1991). Using these screening tools, patients can be identified and referred to the most adequate treatment. In primary hyperventilation, breathing exercises, and relaxation therapy might be recommended. Secondary hyperventilation might require cognitive behavioral therapy with or without a combination of tilt training. Since 67% of patients did not continue tilt training after HV diagnosis and were referred for specific treatment, we hypothesize that primary hyperventilation was present. Primary hyperventilation can induce loss of consciousness because the induced hypocapnia can result in vasoconstriction of the cerebral arterioles and dilatation of the peripheral vessels, contributing to hypotension and cerebral hypoperfusion and a higher chance of loss of consciousness. In secondary hyperventilation, patients with reflex syncope increase the depth of respiration as a compensatory response to the drop in blood pressure in reflex syncope. This causes a reduction in cerebral perfusion and worsens symptoms of reflex syncope (Novak et al., 1998; Norcliffe-Kaufmann et al., 2008; Porta et al., 2008). A recent systematic review showed that yoga is an effective treatment in reducing the number of episodes of (pre)syncope (Abdelazeem et al., 2022). This therapy might be ideal to implement in patients with secondary hyperventilation.

A multidisciplinary approach is useful in patients with RS. After performing the tilt test, patients are often insecure and have many questions. Therefore, collaboration among a cardiologist, physiotherapist, and psychologist is important for effectively supporting patients with RS.

Performing an HVPT, including a consult with a psychologist, is time-consuming and might not always be possible. Another possibility to diagnose HV is the capnography tilt test (CTT), an extension of the tilt test. In 1992, Naschitz et al. (1998) and Naschitz et al. (1997) developed this non-invasive, simple, and inexpensive technique to provide a more detailed evaluation of HV in RS pediatric patients. They performed the tilt test with simultaneous monitoring of the end-tidal PETCO_2_. Martinón-Torres et al. (2001) suggested the inclusion of capnography in tilt protocols in pediatric patients, believing that CTT could improve screening and treatment in the assessment of HV in RS. The study by Lagi et al. (2001) already reported that patients complained of discomfort, fatigue, anxiety, breathing difficulty, and nausea during CTT, even before symptoms or syncope occurred.

### 4.3 Limitations

Our study has several limitations. It is a small, retrospective, monocentric study, making it difficult to determine causality. The monocentric approach makes generalization challenging. Hyperventilation testing was not systematically performed, but only upon clinical suspicion. Future prospective multicenter studies are needed to confirm our results.

We report a reasonable success of tilt training in a contemporary cohort of patients with RS. In patients completing the tilt training program, presyncope and syncope recurrence were low. Concomitant hyperventilation seems prevalent in patients with reflex syncope and warrants a multidisciplinary treatment approach.

## Data Availability

The original contributions presented in the study are included in the article/supplementary material, further inquiries can be directed to the corresponding author.
